# Correction: Nanocellulose/wood ash-reinforced starch–chitosan hydrogel composites for soil conditioning and their impact on pea plant growth

**DOI:** 10.1039/d5ra90004b

**Published:** 2025-01-10

**Authors:** Dure Najaf Iqbal, Zaryab Tariq, Boiz Philips, Ayesha Sadiqa, Muhammad Ahmad, Khairia Mohammed Al-Ahmary, Ijaz Ali, Mahmood Ahmed

**Affiliations:** a Department of Chemistry, The University of Lahore Lahore Pakistan dure.najaf@chem.uol.edu.pk; b Department of Chemistry, Division of Science and Technology, University of Education Lahore 54770 Pakistan mahmood.ahmed@ue.edu.pk; c Department of Chemistry, College of Science, University of Jeddah Jeddah Saudi Arabia; d Centre for Applied Mathematics and Bioinformatics (CAMB), Gulf University for Science and Technology Hawally Kuwait

## Abstract

Correction for ‘Nanocellulose/wood ash-reinforced starch–chitosan hydrogel composites for soil conditioning and their impact on pea plant growth’ by Dure Najaf Iqbal *et al.*, *RSC Adv.*, 2024, **14**, 8652–8664, https://doi.org/10.1039/D3RA08725E.

The authors regret that the coding of the raw data for the FTIR results was incorrect, leading to the generation of inaccurate FTIR spectra in Fig. 3.

The figure and associated text in Section 3.1, FTIR and XRD analysis, should have been:

Because of its random coil shape, CS usually exhibits the amide band at 1700–1600 cm−^1^. The amide band moves to a lower wavenumber (about 1580–1390 cm^−1^) following crosslinking, signifying the creation of new amide bonds with citric acid. The characteristic peaks were observed at 2940–2920 cm^−1^, 1580–1524 cm^−1^, 1395–1390 cm^−1^ and 1150–1143 cm^−1^ for all hydrogel composite samples. This confirms the cross-linking reaction between the amino group (–NH_2_) of CS and the carboxyl group (–COOH) of citric acid. A characteristic peak for all hydrogel composite samples was observed in a region of 1713–1710 cm^−1^, which is accompanied by a strong peak at 1001–1000 cm^−1^ (C–O stretch). This indicated the presence of an ester group in their chemical structure and confirmed the esterification reaction (chemical cross-linking) between the carboxyl group (–COOH) of citric acid and the hydroxyl group (–OH) of SC. Before crosslinking, the citric acid peak is around 1650–1600 cm^−1^ due to its free carboxyl groups. After cross-linking, this peak diminishes or disappears as the carboxyl groups react with chitosan amine groups and the hydroxyl group (–OH) of starch. The FTIR spectrum of the WAC-2 hydrogel composite sample, which contains wood ash as an additive, showed two new peaks: one for carbonate (C–O bending) at 889 cm^−1^ and the other for CO_2_ bending at 662 cm^−1^, which are found in the wood ash.^4^ Two new peaks for ammonium, one for N–H bending at 1625 cm^−1^ and the other at 3211 cm^−1^ due to N–H stretching, were also observed in the FTIR spectrum of the FCC-4 hydrogel sample, which contains NPK fertilizer (ammonium nitrate, ammonium phosphate and potassium chloride) as an additive.
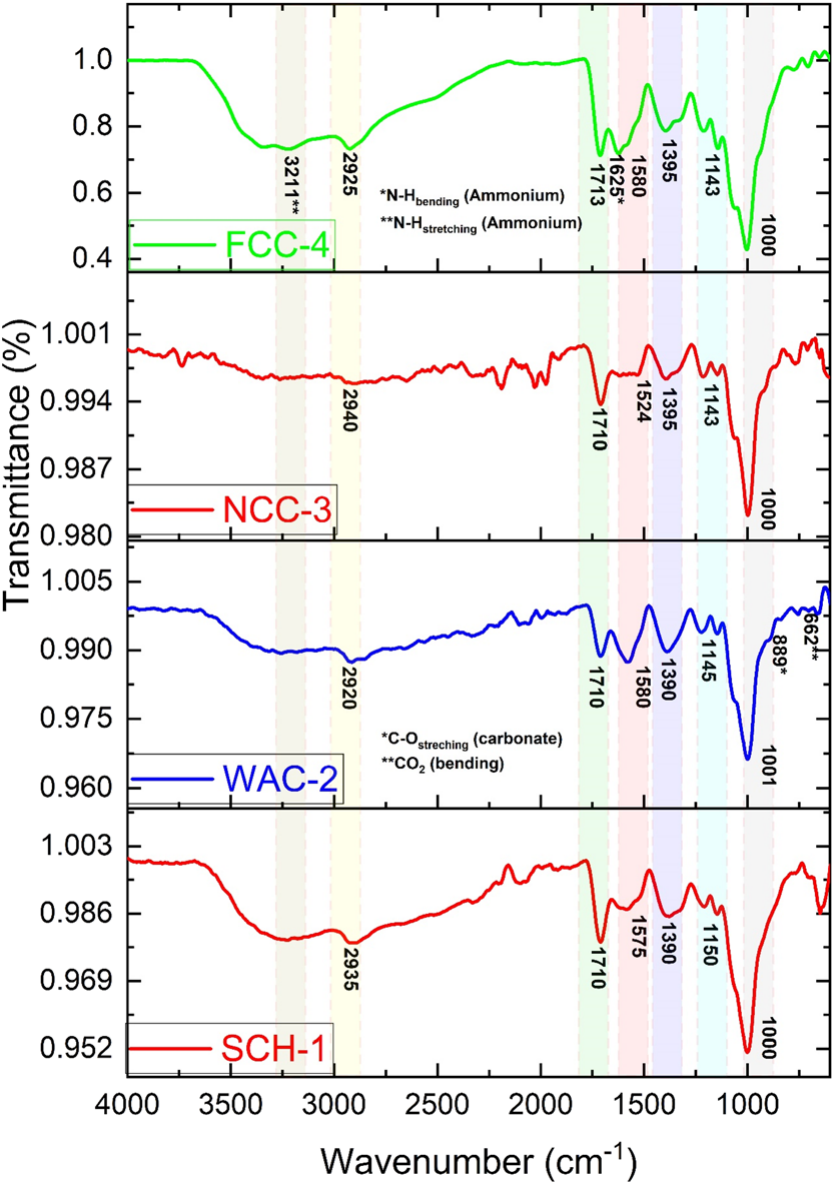


**Fig. 3** FTIR spectra of hydrogel/hydrogel composites.

An independent expert has viewed the corrected Fig. 3 and text, and confirmed that it is consistent with the discussions and conclusions presented.

The Royal Society of Chemistry apologises for these errors and any consequent inconvenience to authors and readers.

